# Systemic inflammation, oxidative damage and neurocognition predict telomere length in a transdiagnostic sample stratified by global DNA methylation levels

**DOI:** 10.1038/s41598-024-62980-6

**Published:** 2024-06-07

**Authors:** Joan Vicent Sánchez-Ortí, Patricia Correa-Ghisays, Vicent Balanzá-Martínez, Diego Macías Saint-Gerons, Ester Berenguer-Pascual, Carlos Romá-Mateo, Víctor M. Victor, Jaume Forés-Martos, Constanza San-Martin, Gabriel Selva-Vera, Rafael Tabarés-Seisdedos

**Affiliations:** 1 INCLIVA - Health Research Institute, Valencia, Spain; 2 TMAP - Evaluation Unit in Personal Autonomy, Dependency and Serious Mental Disorders, University of Valencia, Valencia, Spain; 3 Faculty of Psychology, University of Valencia, Valencia, Spain; 4 Center for Biomedical Research in Mental Health Network (CIBERSAM), Health Institute, Carlos III, Madrid, Spain; 5 Teaching Unit of Psychiatry and Psychological Medicine, Department of Medicine, University of Valencia, Valencia, Spain; 6 VALSME (VALencia Salut Mental i Estigma), University of Valencia, Valencia, Spain; 7 EpiDisease S.L., University of Valencia Scientific Park, Paterna, Valencia, Spain; 8 CIBER de Enfermedades Raras (CIBERER), Health Institute, Carlos III, Madrid, Spain; 9 Department of Physiology, Faculty of Medicine and Dentistry, University of Valencia, Valencia, Spain; 10 Service of Endocrinology and Nutrition, University Hospital Dr. Peset, Valencia, Spain; 11 Foundation for the Promotion of Health and Biomedical Research in the Valencian Region (FISABIO), Valencia, Spain; 12 Department of Physiotherapy, University of Valencia, Valencia, Spain

**Keywords:** DNA methylation, Endocrine system and metabolic diseases, Inflammation, Endocrine system and metabolic diseases, Psychiatric disorders

## Abstract

Epigenetic mechanisms contribute to the maintenance of both type 2 diabetes mellitus (T2DM) and psychiatric disorders. Emerging evidence suggests that molecular pathways and neurocognitive performance regulate epigenetic dynamics in these disorders. The current combined and transdiagnostic study investigated whether inflammatory, oxidative stress, adhesion molecule, neurocognitive and functional performance are significant predictors of telomere dynamics in a sample stratified by global DNA methylation levels. Peripheral blood inflammation, oxidative stress and adhesion molecule biomarkers and neurocognitive function were assessed twice over a 1-year period in 80 individuals, including 16 with schizophrenia (SZ), 16 with bipolar disorder (BD), 16 with major depressive disorder (MDD), 15 with T2DM, and 17 healthy controls (HCs). Leukocyte telomere length (LTL) was measured by qRT-PCR using deoxyribonucleic acid (DNA) extracted from peripheral blood samples. A posteriori, individuals were classified based on their global methylation score (GMS) at baseline into two groups: the below-average methylation (BM) and above-average methylation (AM) groups. Hierarchical and k-means clustering methods, mixed one-way analysis of variance and linear regression analyses were performed. Overall, the BM group showed a significantly higher leukocyte telomere length (LTL) than the AM group at both time points (*p* = 0.02; η^2^p = 0.06). Moreover, the BM group had significantly lower levels of tumor necrosis factor alpha (TNF-α) (*p* = 0.03; η^2^p = 0.06) and C-reactive protein (CRP) (*p* = 0.03; η^2^p = 0.06) than the AM group at the 1-year follow-up. Across all participants, the regression models showed that oxidative stress (reactive oxygen species [ROS]) (*p* = 0.04) and global cognitive score [GCS] (*p* = 0.02) were significantly negatively associated with LTL, whereas inflammatory (TNF-α) (*p* = 0.04), adhesion molecule biomarkers (inter cellular adhesion molecule [ICAM]) (*p* = 0.009), and intelligence quotient [IQ] (*p* = 0.03) were significantly positively associated with LTL. Moreover, the model predictive power was increased when tested in both groups separately, explaining 15.8% and 28.1% of the LTL variance at the 1-year follow-up for the AM and BM groups, respectively. Heterogeneous DNA methylation in individuals with T2DM and severe mental disorders seems to support the hypothesis that epigenetic dysregulation occurs in a transdiagnostic manner. Our results may help to elucidate the interplay between epigenetics, molecular processes and neurocognitive function in these disorders. DNA methylation and LTL are potential therapeutic targets for transdiagnostic interventions to decrease the risk of comorbidities.

## Introduction

Over recent years, the progressively increasing body of research on mental health problems has highlighted the significant role of nongenetic factors, such as environmental interactions and epigenetics^1^. Epigenetic processes involve regulating gene expression through deoxyribonucleic acid (DNA) chemical modifications, histone tail modifications and variants, and noncoding ribonucleic acid (RNA); these mechanisms all contribute to silencing gene expression to produce a significant change in the cellular phenotype that can be transmitted postmitotically in response to environmental factors^2^. Decreased DNA methylation increases gene expression, while DNA hypermethylation is associated with reduced gene expression^3^. Alterations in these epigenetic mechanisms are implicated in the pathogenesis of many human diseases, including psychiatric disorders^4^.

The comorbidity of type 2 diabetes mellitus (T2DM) and various psychiatric disorders is well established. According to recent epidemiological reviews, the prevalence of T2DM is higher in individuals with major depressive disorder (MDD), bipolar disorder (BD) and schizophrenia (SZ) than that in the general population, and individuals with T2DM are at increased risk of developing psychiatric disorders^5^. Currently, T2DM and psychiatric disorders are known to share several pathogenic pathways, including an elevated inflammatory state, oxidative stress damage, aberrant cellular adhesion and neurocognitive impairment, which influence each other^6^. The complex interactions of these pathways are reflected in clinical phenotypic expression that is induced by altered gene regulation^7^. In fact, a significant body of evidence shows that epigenetic mechanisms contribute extensively to the cooccurrence of these disorders^8^. Inflammatory activity, in turn, is thought to promote DNA methylation, and a recent study showed that oxidative stress contributes to DNA damage and methylation in early human life, thus leading to either pathological neurodevelopment or behavioural dysfunction^9,10^.

Leukocyte telomere length (LTL) is involved in chromosomal stability and prevents DNA damage^11^. Emerging evidence has suggested that dysfunctional DNA methylation is strongly associated with a shortened LTL, thus increasing the risks of developing chronic disorders and overall mortality^12^. For instance, it was observed that LTLs were significantly shorter in individuals with MDD^13^, BD^14^, SZ^15^ and T2DM^16^ than in healthy controls (HCs). Likewise, studies have shown that telomere dysfunction has a reciprocal causation relationship with potential changes in inflammatory mechanisms and oxidative stress pathways^17,18^. Concomitantly, from a functional perspective, there is evidence that LTL contributes to the maintenance of neurocognitive performance and may explain, at least in part, different trajectories of neurocognitive functioning^19^. There is also consistent evidence that LTL is essential to a healthy lifestyle^20^ and therapeutic responses across psychiatric disorders^21^. However, the relationships between epigenetics (in particular, DNA methylation), inflammatory mechanisms, oxidative stress, and telomere dynamics remain unknown in people with T2DM and psychiatric disorders. This research gap hampers better understanding whether inflammatory, oxidative stress, adhesion molecule, neurocognitive, and functional performance markers are significant predictors of telomere dynamics. This study aimed to identify markers valid for telomere length alteration from a combined, transdiagnostic sample stratified by global DNA methylation levels.

## Materials and methods

### Study design and ethical considerations

This study is part of a project aimed at identifying and validating peripheral biomarkers for neurocognitive deficits in MDD, BD, SZ and T2DM. This prospective and comparative cohort project was conducted between 2018 and 2020 and aimed to investigate the association and evolution of certain peripheral blood biomarkers and neurocognitive impairments in a unique longitudinal cohort of individuals with somatic and psychiatric disorders. Demographic and clinical data, neurocognitive and functional data, and levels of peripheral blood biomarkers were collected at baseline (T1) and after one year (T2). Individuals with severe mental illnesses (SMI) were recruited from mental health units (MHUs) in several towns in the province of Valencia, Spain (Gandía, Foios, Catarroja, Paterna, and Sagunto), the psychiatry outpatient clinic and endocrinology department of the University Hospital Dr. Peset, and the Miguel Servet MHU in Valencia City. HCs were residents of the same areas as the individuals with SMI. Participants were demographically matched. All participants provided informed consent after the study procedures were fully explained. This study was approved by the Ethical Committee for Research with Medicines of the University Clinical Hospital of Valencia (reference no. 2017/185) and the study was conducted in accordance with the ethical principles of the Declaration of Helsinki. For this study, only those variables related to the present study aims were included in the analyses.

### Participants

SZ, BD, and MDD were diagnosed according to the criteria of the Diagnostic and Statistical Manual of Mental Disorders 5th edition [DSM-5]^22^. T2DM was diagnosed based on the Standards of Care criteria of the American Diabetes Association^23^. Participants with MDD and BD were required to meet the remission criteria^24^ of an acute affective episode, and individuals with SZ were required to be clinically stable^25^. Individuals with T2DM were required to be free of severe diabetic neuropathy and kidney disease (serum creatinine < 1.5 mg/dl). For recruitment as HC, the exclusion criteria were physical illness, pharmacological treatments, and family history of SMI in first-degree relatives. The ability to understand the study procedures and willingness to give written consent were required for participation. The general exclusion criteria for all groups included current hospitalization, documented cognitive impairment not secondary to psychiatric disorder (intellectual disability or major neurocognitive disorder, i.e., dementia), disability or other inability that prevented understanding of the protocol, current substance abuse disorder (except for nicotine), pregnancy, intake of steroids, corticosteroids, antioxidants, antibiotics, and immunologic therapies, fever over 38 °C, and history of vaccination within 4 weeks of the evaluation. The same inclusion and exclusion criteria were used at T1 and T2. The participants were matched according to their geographic region, gender, age, and years of education.

### Clinical and neuropsychological assessments

The assessments were conducted by the same experienced psychologists and psychiatrists of the research group. Sociodemographic data, including sex, age, years of education, and motor laterality (defined as manual, ocular and crural dominance), were collected.

Clinical evaluations were conducted using the following instruments: (i) the Kaplan-Feinstein Scale (KFS)^26^, (ii) Charlson Comorbidity Index (CCI)^27^, (iii) 17-item Hamilton Rating Scale for Depression (HRSD)^28^, (iv) Young Mania Rating Scale (YMRS)^29^, and (v) Positive and Negative Syndrome Scale (PANSS)^30^. The number of people who were current tobacco consumers and their breath carboxyhemoglobin (COHb) levels, a validated measure of smoke exposure, were collected. Anthropometric variables height (m) and body weight (kg) were measured by calibrated scales. Body mass index (BMI) was calculated as kg/m^2^^31^. The total number of prescribed psychopharmacological medications was also recorded.

Cognitive performance was evaluated using a comprehensive battery of neuropsychological tests and subtests previously used by our group (CIBERSAM-G24). Eight cognitive domains were assessed: (i) general intelligence as reflected by the premorbid intelligence quotient (IQ), which was calculated using the Wechsler Adult Intelligence Scale III edition (WAIS-III)^32^ Vocabulary subtest and is considered a classical measure of the level of intelligence before the onset of a mental disorder^33^; (ii) verbal learning and memory, which was measured with the Complutense Verbal Learning Test (TAVEC)^34^ total immediate recall, short-term free recall and long-term free recall scores, (iii) cognitive flexibility measured with the Stroop Color and Word test (SCWT)^35^ color/word subtest and, Wisconsin Card Sorting Test (WCST)^36^ categories completed and perseverative errors scores, (iv verbal fluency determined as the FAS test (phonemic fluency) score and Animal Naming test (semantic fluency) score^37^, (v) working memory evaluated by the Trail Making Test (TMT)^37^ Part B and, WAIS-III digit span backward subtest, (vi) short-term memory measured as the TAVEC immediate recall of the first learning trial and immediate recall of the interference list scores and, WAIS-III digit span forward subtest; (vii) visual memory determined by the Rey-Osterrieth Complex Figure Test (ROCFT)^38^ recall two minutes (fRey2) and 20 min after the copy (fRey20), and, (viii) processing speed as evaluated with the finger tapping test (FTT)^37,39^ left unimanual, right unimanual, left bimanual, right bimanual and the four average scores, WAIS-III digit symbol coding subtest, SCWT color and word subtests and TMT Part A. A global cognitive score (GCS) was calculated by averaging the eight cognitive domain scores.

Functional performance was evaluated using (i) the Functional Assessment Short Test (FAST)^40^, (ii) the Short Form-36 Health Survey questionnaire (SF-36)^41^, and (iii) the World Health Organization Quality of Life-Brief scale (WHO-QoL-Bref)^42^. A global functional score (GFS) was calculated by averaging the total scores on the three scales.

### Determination of biomarkers in peripheral blood

Venous blood extraction was performed, and the serum and plasma samples were kept in a freezer at − 80 °C.

#### Cytokine measurement

Serum cytokine concentrations were determined using Luminex® X-MAP technology (Luminex Corp., Austin, TX, USA). The following cytokines were analyzed: interleukin 6 (IL-6), interleukin 10 (IL-10) and tumor necrosis factor alpha (TNF-α). Sample processing and data analysis were performed according to the manufacturer's instructions. C-reactive protein (CRP) levels were determined by an immunonephelometric assay (Behring Nephelometer II, Dade Behring, Inc., Newark, DE, USA). After 12 h of overnight fasting, blood samples were taken from 8:00 to 10:00 a.m. and centrifuged (1.500 g, 10 min, 4 °C) to separate serum or plasma prior to determining biochemical and molecular parameters.

#### Oxidative stress and mitochondrial metabolism measurement

The oxidative stress of leukocytes was evaluated using fluorimetry techniques in a fluoroscan system (Synergy MX). A total of 100,000 cells were plated in each well of a 96-well plate and incubated for 30 min at 37 °C with the corresponding fluorochrome: dichlorofluorescein diacetate indicated reactive oxygen species (ROS) production (485 nm excitation, 535 nm emission), MitoSOX measured mitochondrial ROS (mROS) (510 nm excitation, 580 nm emission), tetramethylrodamin methyl ester (552 nm excitation, 574 nm emission) assessed mitochondrial membrane potential, nonylacridin orange mitochondrial mass (495 nm excitation, 519 nm emission), and 5-chloromethylfluorescein diacetate measured intracellular glutathione (492 nm excitation, 517 nm emission). We used the monocyte cell line U-937 as an internal control to avoid the possible confounds of fluctuating fluorescence with time. Serum lipid peroxidation levels were measured using a commercial thiobarbituric acid reactive substances (TBARS) kit according to the manufacturer's instructions (Olympus, Hamburg, Germany).

#### Adhesion molecule measurement

A Luminex 200 flow analyzer system was employed to analyze adhesion molecules in serum (Austin, TX, USA). Citrated blood samples were incubated with dextran (3%) for 45 min to isolate human polymorphonuclear leukocytes (PMNs). The supernatant was added to Ficoll-Hypaque (GE Healthcare, Barcelona, Spain) and centrifuged for 25 min at room temperature at 650 g. Lysis buffer was added to the erythrocytes remaining in the pellet, which was incubated at room temperature for 5 min and then centrifuged at 240 g for 5 min. PMNs were washed twice and resuspended at 37 °C in Hanks' balanced salt solution (HBSS; Sigma Aldrich, MO). Scepter 2.0 cell counters (Millipore, MA, USA) were employed to count cells.

#### Telomere measurement

The LTL was measured using DNA extracted from peripheral blood samples. The fraction of peripheral mononuclear blood cells was obtained using BD Vacutainer® CPT™ Mononuclear Cell Preparation Tube—Sodium Citrate (BD Biosciences, NJ, USA). Each tube was centrifuged at 1800 rpm for 25 min to separate plasma from red and white cells, after which the fraction corresponding to white cells was centrifuged at 2500 rpm for 10 min at 4 °C; the supernatant was discarded, and pellets were washed with PBS. Finally, dry pellets were stored at − 80 °C until DNA extraction. DNA was isolated using the Qiagen DNeasy Blood & Tissue Kit (Hilden, Germany) following the manufacturer's instructions for cellular material. Then, telomere length was measured and compared as the telomere-to-single copy gene ratio by quantitative real-time PCR. Primer sequences, the concentrations of the telomere and 36B4 gene, the PCR conditions, and quantification protocols described by Cawthon^43^ were used with some modifications. Each sample was analyzed in triplicate using 10 ng of DNA, 7 µL of H2O, 10 µL of master mix, and 2 µL oligos per sample. For each standard curve, a reference DNA sample (1,691,112, Roche Diagnostics, Barcelona, Spain) was diluted serially in water at a 1:10 dilution ratio, where the range of concentrations started from 200 to 0.2 ng in 1 µL. The thermal cycling profile was as follows: one cycle of 15 min at 95 °C, followed by 45 cycles of 10 s at 95 °C, 5 s at 55 °C and 11 s at 72 °C.

### Methylation analysis using EPIC 850 K methylation arrays

500 ng of genomic DNA (purified as described above) was bisulfite converted using the EZ-96 DNA Methylation Kit (Zymo Research, Irvine, CA, USA) following the manufacturer’s recommendations for Infinium EPIC methylation assays. Afterwards, 4 µL of bisulfite-converted DNA were used following the Illumina Infinium HD Methylation Assay protocol as previously described^44^. Specifically, DNA methylation analysis was performed with the Infinium MethylationEPIC850K v1.0 BeadChip (Illumina Inc, San Diego, CA, USA), used to analyze ~ 850,000 CpGs. This array covers 99% of genes described and 95% of CpG islands, including data from projects such as ENCODE and FANTOM5. First, we performed a whole genome amplification step followed by enzymatic endpoint fragmentation, precipitation, and resuspension. Afterwards, processed samples were hybridized on Infinium MethylationEPIC v1.0 BeadChips at 48 °C for 16 h. Unhybridized and non-specifically hybridized DNA were washed away. To this chemical reaction we next added a single nucleotide extension, using the hybridized bisulfite-treated DNA as a template, nucleotides labeled with biotin (ddCTP and ddGTP), and 2,4-dinitrophenol (ddATP and ddTTP). After the single base extension, several repeated rounds of staining were performed with a combination of antibodies that differentiated DNP and biotin by fixing them with different fluorophores. Finally, the BeadChip was washed and protected for scanning on the Illumina HiScan SQ scanner (Illumina Inc, San Diego, CA, USA).

#### Bioinformatics analysis

The minfi R-package^45^ was used to read raw IDAT files obtained from the Illumina EPIC array, assess their quality, and perform the normalization and the exclusion of probes that might interfere in subsequent analysis^46^. DNA methylation probes were filtered to remove those with poor detection *p*-values (< 0.01) in any sample, those that matched with previously described specific SNP positions, sex-related probes (X & Y chromosome) and those described as multiple reactive probes^47^.

#### Clustering DNA methylation data

**Figure 1:**
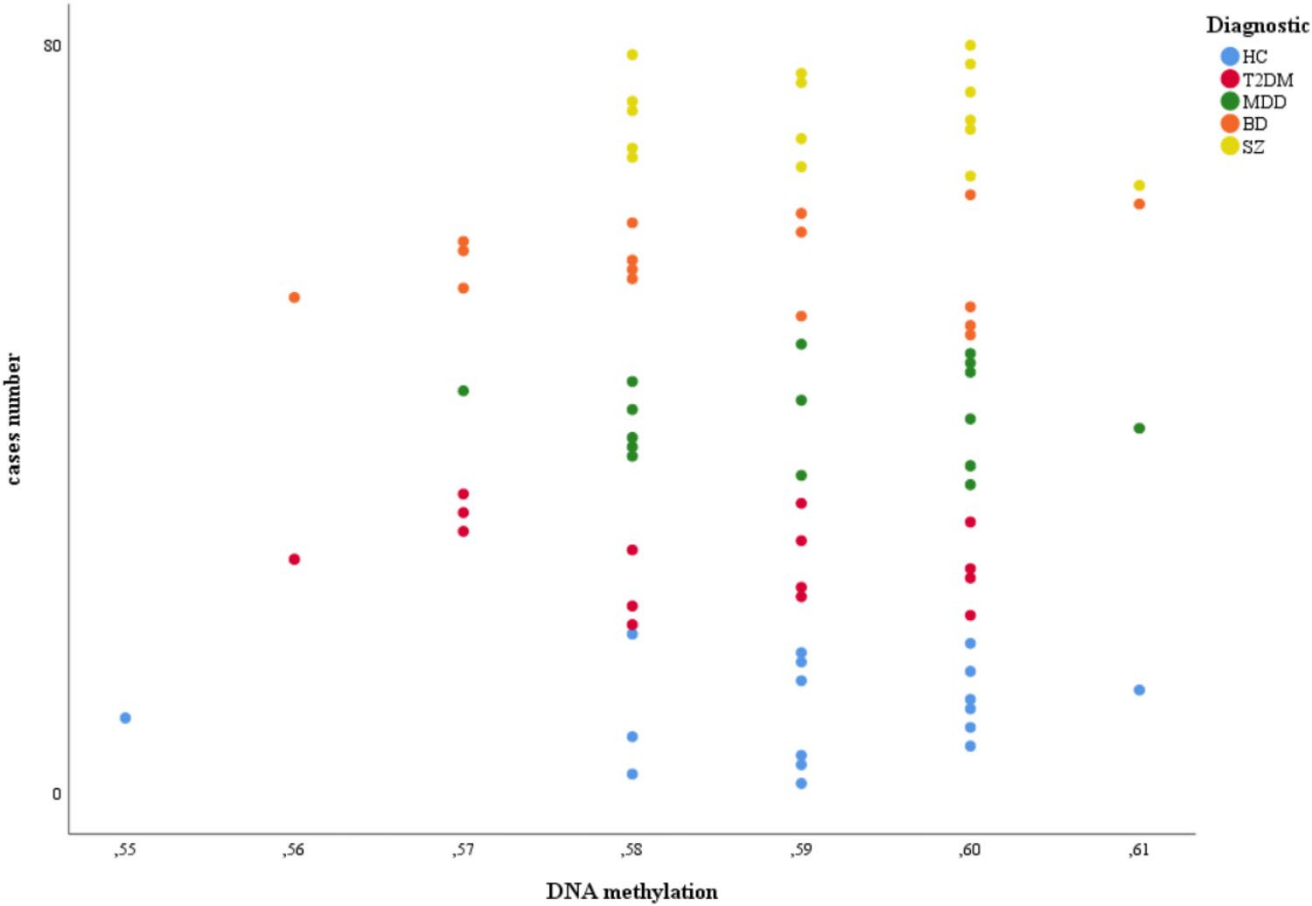
Global DNA methylation in the whole sample. HC = Healthy controls, T2DM = type 2 diabetes mellitus, MDD = major depressive disorder, BD = bipolar disorder, SZ = schizophrenia.

Intensity values were converted to beta values (β-values). Then, the global methylation score (GMS) was measured as the β-value. The β-value results in a number between 0 and 1; a value of zero indicated that all copies of the CpG site in the sample were completely unmethylated and a value of one indicated that every copy of the site was methylated. Global DNA methylation reflected the methylation status of the total genomic content within the whole sample^48^ (Fig. 1).

There are no established biological thresholds for the categorization of global methylation, so hierarchical and k-means cluster analyses were performed. These methods merge individuals based on distance functions; this distance is measured as a function of all pairs of observations from different clusters. In our analyses, we used an average linkage function. Moreover, observations were merged to increase the classification likelihood. Using GMS data measured at CpG regions, these clustering methods generated a two-group model. Moreover, all data were verified to have a normal distribution. We assumed that within each group, the methylation values followed the same distribution for each person independently. A posteriori, individuals were classified into two groups based on their global methylation score (GMS) at baseline: the below-average methylation (BM) and above-average methylation (AM) group.

### Statistical analyses

Data were analyzed using Statistical Package for Social Sciences (SPSS) version 26.0 for Windows^49^. The hierarchical clustering method was used in the data set to produce two clusters across 80 clinical cases, which were explored descriptively and named by examining the characteristics of commonalities within each cluster. Finally, k-means clustering was used to assign individual participants to the cluster they fit most closely. For all cluster analyses, the reference variable was the GMS measured from the β-value. Descriptive analyses were conducted using Student's t tests for independent samples for continuous variables and chi-square tests for categorical variables. Moreover, the possible relationship between age and LTL was previously tested. For this purpose, a Pearson correlation test was applied, which showed non-significant results (r = − 0.20; *p* = 0.07). The between-group differences in neurocognitive and functional performance and biomarker levels at T1 and T2 and their evolution over time were assessed using a mixed ANOVA. The sample size was calculated using Ene 2.0 software, which estimated that twenty individuals for each sample group was sufficient to ensure the representativeness. Normality was assumed for all continuous variables because the sample was sufficiently representative of the target population; this assumption was statistically verified using Shapiro–Wilk test, guaranteeing that the variable groups for T1 and T2 could be assessed using ANOVA. A post hoc analysis with a Bonferroni-corrected pairwise t test and Mann‒Whitney U test was performed to examine the differences between groups. The effect size was calculated with partial eta-squared (η^2^p), and the following values were taken as references: small ≈ 0.02; moderate ≈ 0.15; and large ≈ 0.35. The raw scores obtained for IQ, GCS and GFS were transformed into Z scores. For the calculation of the Z scores, the mean and standard deviation of the individuals with BM at T1 were taken as reference values. To test the predictive capacity of outcomes at baseline for explaining the variance in LTL over time, a linear regression analysis was performed using a predictive model that included biomarker levels and neurocognitive and functional performance scores that were significant for the whole sample. A posteriori, the transdiagnostic predictive model was checked in each group. Other variables relevant to LTL were not included because they were not relevant to the aim of this study. This was tested by a double check: (1) previous literature reviewed and (2) testing of each of the socio-demographic and clinical variables separately and together in regression models. For all analyses, *p* < 0.05 was considered the threshold for statistical significance. The procedure to create the predictive models was as follows: first, a predictive analysis was performed using biomarker levels, IQ, GCS, and GFS individually, and then predictive models were generated that included and combined the statistically more powerful variables; finally, the optimal predictive combination was obtained. No more than five variables were included in each model, thus guaranteeing the correct performance of the analysis.

## Results

### Clustering of individuals from DNA methylation

**Figure 2:**
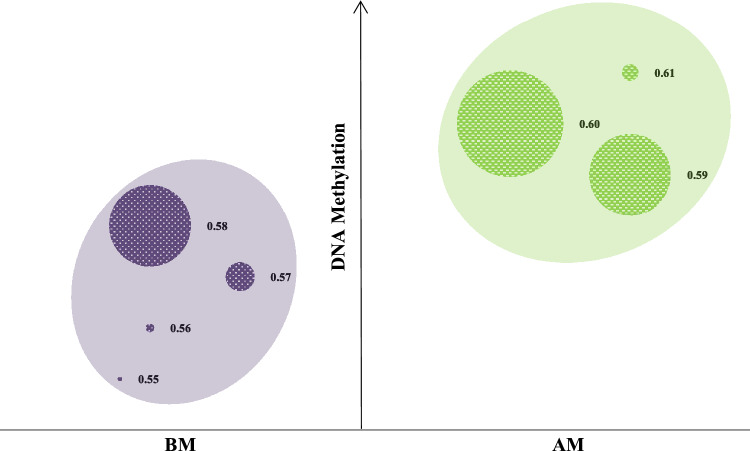
Distribution of the individuals in each group from global DNA methylation. BM = below-average methylation, AM = above-average methylation.

After hierarchal clustering of the 80 clinical cases, two main clusters were observed. The BM cluster primarily consisted of individuals with lower global DNA methylation; in contrast, the AM cluster contained individuals with higher global DNA methylation. These results were found to be stable by k-means clustering. Figure 2 shows the distribution of the individuals into each group accounting for GMS. When comparing the two main clusters (BM and AM), the median GMS was significantly higher in the AM cluster than in the BM cluster (*p* = 0.002), whereas the pattern of DNA methylation was similar among the individuals who composed each group (*p* = 0.67).

### Sample description

At T1, the sample consisted of 80 persons, including 16 with SZ, 16 with BD, 16 with MDD, 15 with T2DM, and 17 HCs. The total sample was classified into two groups: 30 in the BM group (SZ = 5, BD = 8, MDD = 6, T2DM = 7 and HC = 4) and 50 in the AM group (SZ = 11, BD = 8, MDD = 10, T2DM = 8 and HC = 13).

Thirteen participants were lost to follow-up at T2 (retention rate: 83.7%). The sample consisted of 30 individuals in the BM group (SZ = 5, BD = 8, MDD = 6, T2DM = 7 and HC = 4) and 37 individuals in the AM group (SZ = 10, BD = 6, MDD = 7, T2DM = 6 and HC = 8).

A summary of the sociodemographic and clinical characteristics of the participants is presented in Table 1. Women represented approximately half of the total sample (46.2%). The mean age of the whole sample was 46.4 (SD: 12.1) years. All sociodemographic and clinical variables were similar across both groups.

**Table 1:** Sociodemographic and clinical characteristics of the sample at T1.

Variables^a^	BM	AM	Statistical analyses
	(*n* = 30)	(*n* = 50)	t or χ^2^(*p*)^gh^
Sociodemographic			
Sex^b^	12(40%)	25(50%)	0.7(*p* = 0.38)
Age	48.8(12.7)	44.1(11.5)	1.6(*p* = 0.10)
Years of education	12.9(5.2)	13.2(4.3)	0.2(*p* = 0.78)
Laterality^c^	27(90%)	43(86%)	0.3(*p* = 0.84)
Clinical			
Tobacco^d^	10(33%)	17(34%)	0.1(*p* = 0.95)
COHb	0.9(1.2)	0.9(1.1)	0.2(*p* = 0.78)
BMI	28.4(4.3)	30.2(6.3)	1.3(*p* = 0.19)
SMI	19(63%)	29(58%)	3.2(*p* = 0.51)
KFS	1.4(1.8)	0.8(1.5)	1.6(*p* = 0.11)
CCI	1.0(1.4)	0.7(1.2)	1.0(*p* = 0.27)
HRSD^e^	6.0(7.3)	6.0(6.0)	0.3(*p* = 0.97)
YMRS^e^	2.2(3.0)	1.5(2.8)	0.9(*p* = 0.34)
PANSS-T^e^	34.8(8.3)	39.7(16.9)	1.4(*p* = 0.14)
Psychiatric medicines^f^	2.3(2.1)	1.7(1.8)	1.3(*p* = 0.18)

^a^Expressed as mean(standard deviation) except when indicated, ^b^female n(%),^c^right-handers n(%),^d^yes n(%), ^e^lower scores represent a better outcome, ^f^number. ^g^t-test for independent samples. ^h^Chi-squared test. Abbreviations: T1 = Time 1, BM = below-average methylation, AM = above-average methylation, COHb = carboxihemoglobina, KFS = kaplan-feinstein scale, CCI = charlson comorbidity index, BMI = body mass index, SMI = severe mental illness, HDRS = hamilton rating scale for depression, YMRS = young mania rating scale, PANSS = positive and negative syndrome scale, T = total, NS = Not Significant. (NS = *p* > 0.05; *p ≤ 0.05; **p ≤ 0.01; ***p ≤ 0.001; ****p ≤ 0.0001).

### Between-group comparison of serum blood biomarker levels and neurocognitive and functional performance

Serum blood biomarker levels and neurocognitive and functional performance at T1 and T2 for both groups are shown in Table 2. Overall, the BM group showed a significantly higher LTL than the AM group at T1 (*p* < 0.05; η^2^p = 0.06), and similar findings were observed at T2 (*p* < 0.05; η^2^p = 0.06). However, individuals in the BM group had significantly lower levels of TNF-α and CRP at T2 compared to those in the AM group (*p* < 0.05; η^2^p = 0.06). For all comparisons, the effect size ranged from small to moderate. Neurocognitive and functional performance scores were similar in both groups. Within-group differences over time were not significant.

**Table 2:** Serum blood biomarkers, cognition and functional performance at T1 and T2.

Variables^a^	BM		AM		Statistical analyses					
	T1	T2	T1	T2	T1	η^2^p^d^	T2	η^2^p^d^	T1-T2	η^2^p ^d^
	(*n* = 30)	(*n* = 30)	(*n* = 50)	(*n* = 37)	F(*p*)^c^		F(*p*)^c^		F(*p*)^c^	
Inflammatory markers										
IL-6	3.0(2.8)	3.9(1.2)	2.6(2.1)	3.2(1.9)	0.4(*p* = 0.49)		0.5(*p* = 0.47)		2.2(*p* = 0.14)	
IL-10	20.8(22.3)	13.3(16.9)	28.4(24.3)	10.8(14.4)	1.6(*p* = 0.13)		1.2(*p* = 0.17)		2.0(*p* = 0.09)	
TNF-α	8.7(3.0)	9.7(2.3)	9.8(2.3)	11.2(3.1)	3.7(*p* = 0.06)		4.5(*p* = 0.03)	0.06	0.2(*p* = 0.69)	
CRP	3.7(4.8)	2.5(2.9)	3.8(5.6)	4.6(4.6)	1.1(*p* = 0.24)		4.3(*p* = 0.03)	0.06	0.3(*p* = 0.56)	
Oxidative stress markers										
GSH	219.1(79.0)	220.4(109.3)	200.6(119.7)	174.5(92.9)	0.1(*p* = 0.89)		0.2(*p* = 0.75)		0.9(*p* = 0.32)	
ROS	152.4(113.9)	325.2(174.8)	129.8(70.3)	285.6(155.9)	0.7(*p* = 0.38)		0.9(*p* = 0.34)		2.3(*p* = 0.13)	
mROS	178.0(80.2)	209.5(91.7)	170.6(68.8)	207.4(99.9)	0.9(*p* = 0.32)		0.1(*p* = 0.82)		1.1(*p* = 0.24)	
SOD	104.6(49.6)	104.2(38.1)	109.4(52.3)	101.9(33.7)	0.3(*p* = 0.54)		0.1(*p* = 0.86)		1.2(*p* = 0.26)	
ΔΨm	52.7(30.0)	53.8(46.2)	52.9(61.2)	38.3(28.6)	0.1(*p* = 0.98)		0.2(*p* = 0.72)		2.4(*p* = 0.12)	
Adhesion molecules										
ICAM	111.6(72.1)	101.6(42.6)	107.3(60.6)	116.3(42.0)	0.1(*p* = 0.83)		0.1(*p* = 0.88)		0.2(*p* = 0.70)	
VCAM	600.8(165.9)	828.9(256.8)	571.2(162.7)	767.7(230.9)	0.1(*p* = 0.84)		0.4(*p* = 0.55)		0.6(*p* = 0.43)	
PSEL	119.5(34.8)	117.8(44.2)	125.3(36.6)	111.5(40.8)	0.8(*p* = 0.41)		0.2(*p* = 0.66)		2.4(*p* = 0.12)	
MPO	608.2(386.6)	651.9(394.2)	560.1(290.2)	628.5(445.0)	0.1(*p* = 0.84)		0.1(*p* = 0.98)		0.9(*p* = 0.34)	
Relative telomere length										
T/S ratio	1.4(0.5)	1.1(0.3)	1.1(0.3)	1.0(0.3)	5.0(*p* = 0.02)	.06	4.6(*p* = 0.02)	0.06	0.2(*p* = 0.63)	
Neurocognitive and functional performance										
IQ^b^	0.0(1.0)	0.5(1.0)	0.1(1.0)	0.8(0.9)	0.2(*p* = 0.61)		0.5(*p* = 0.58)		0.7(*p* = 0.38)	
GCS^b^	0.0(1.0)	0.1(0.9)	0.2(1.0)	0.1(1.1)	1.2(*p* = 0.26)		1.1(*p* = 0.29)		2.2(*p* = 0.13)	
GFS^b^	0.0(1.0)	0.0(1.1)	0.2(0.9)	0.2(0.9)	1.2(*p* = 0.27)		1.2(*p* = 0.26)		0.4(*p* = 0.54)	

^a^Expressed as mean(standard deviation). ^b^Z-Scores expressed as mean(standard deviation). ^c^ANOVA. ^d^Partial Eta-Squared (η^2^p). Abbreviations: T1 = time 1, T2 = time 2, BM = below-average methylation, AM = above-average methylation, IL-6 = interleukin-6, IL-10 = interleukin-10, TNF-α = tumor necrosis factor alpha, CRP = c-reactive protein, GSH = glutathione, ROS = reactive oxygen species, mROS = mitochondrial reactive oxygen species, SOD = superoxide dismutase, ΔΨm = mitochondrial membrane potential, CAM = cellular adhesion molecule, I = inter, V = vascular, PSEL = pselectin, MPO = myeloperoxidase, T/S ratio = relative telomere to single copy gene ratio, IQ = intelligence quotient, GCS = global cognitive score, GFS = global functional score, NS = not significant. (NS = *p* > 0.05; *p ≤ 0.05; **p ≤ 0.01; ***p ≤ 0.001; ****p ≤ 0.0001). Effect size (η^2^p: small ≈ 0.02; moderate ≈ 0.15; large ≈ 0.35).

### The ability of blood biomarker levels and neurocognitive and functional performance scores at T1 to predict telomere length at T2

**Figure 3:**
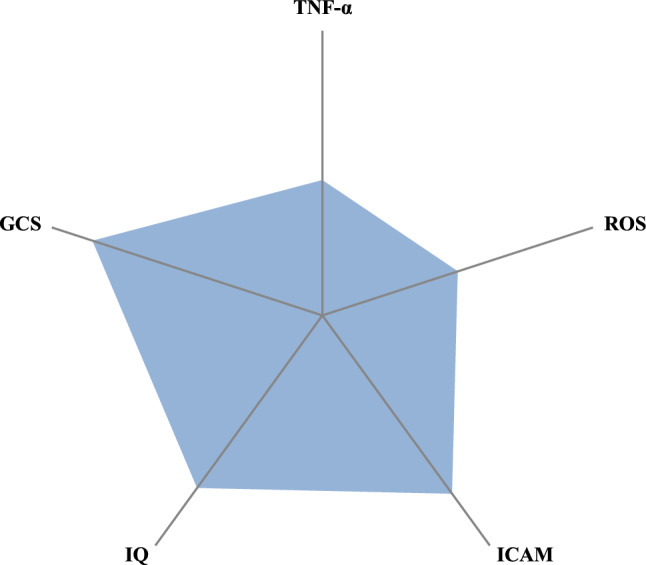
Variance explained by blood serum biomarkers and neurocognitive performance in whole sample. TNF-α = tumor necrosis factor alpha, ROS = reactive oxygen species, CAM = cellular adhesion molecule, I = inter, IQ = intelligence quotient, GCS = global cognitive score.

The results of the relative contributions of blood biomarker levels and neurocognitive and functional performance scores at T1 to explaining the LTL at T2 are shown in Table 3. For the whole sample, the combination of proinflammatory (TNF-α) and oxidative stress biomarker levels (ROS), adhesion molecules (intercellular adhesion molecule [ICAM]) and neurocognitive performance (IQ and GCS) significantly predicted LTL at T2 and explained 14.4% of the variance (Fig. 3). Moreover, the model predictive power was increased when tested in both groups separately and explained 15.8% and 28.1% of the LTL variance at T2 for the AM and BM groups, respectively.

**Table 3:** Predictive outcomes at T1 of relative telomere length at T2.

Dependent variables at T2	Predictors at T1 associated	*β*	95% CI	t(*p*)	Percent of variance explained (adjusted *R*^2^)
Whole sample					
T/S Ratio	TNF-α	0.19	0.00 to 0.05	1.9(*p* = 0.04)	14.4
	ROS	− 0.20	− 0.002 to 0.001	1.9(*p* = 0.04)	
	ICAM	0.31	0.00 to 0.003	2.8(*p* = 0.009)	
	IQ	0.30	0.00 to 0.19	2.1(*p* = 0.03)	
	GCS	− 0.34	− 0.19 to − 0.01	2.6(*p* = 0.02)	
BM group					
T/S Ratio	TNF-α	0.27	0.005 to 0.07	1.8(*p* = 0.04)	28.1
	ROS	− 0.33	− 0.002 to 0.001	2.0(*p* = 0.03)	
	ICAM	0.50	0.001 to 0.004	3.1(*p* = 0.004)	
	IQ	0.28	− 0.06 to 0.19	2.0(*p* = 0.04)	
	GCS	− 0.25	− 0.18 to 0.07	1.8(*p* = 0.02)	
AM group					
T/S Ratio	TNF-α	0.23	− 0.02 to 0.06	1.7(*p* = 0.05)	15.8
	ROS	− 0.24	− 0.004 to 0.002	1.8(*p* = 0.04)	
	ICAM	0.28	− 0.001 to 0.002	1.9(*p* = 0.04)	
	IQ	0.59	0.03 to 0.29	2.6(*p* = 0.01)	
	GCS	− 0.66	− 0.30 to − 0.05	2.8(*p* = 0.007)	

T1 = time 1, T2 = time 2, CI = Confidence interval, BM = below-average methylation, AM = above-average methylation, T/S ratio = relative telomere to single copy gene ratio, TNF-α = tumor necrosis factor alpha, ROS = reactive oxygen species, CAM = cellular adhesion molecule, I = inter, IQ = intelligence quotient, GCS = global cognitive score, NS = not significant. (NS = *p* > 0.05; *p ≤ 0.05; **p ≤ 0.01; ***p ≤ 0.001; ****p ≤ 0.0001).

## Discussion

To our knowledge, this is the first study that assessed whether inflammatory, oxidative stress and adhesion molecule biomarker levels, together with neurocognitive and functional performance scores, are significant predictors of telomere dynamics in a transdiagnostic sample stratified by global DNA methylation.

Our findings show that lower levels of global DNA methylation were associated with longer LTLs across individuals with T2DM and severe mental disorders. Likewise, the results partly support a relationship between proinflammatory factors and global DNA methylation. Regarding telomere dynamics, a set of peripheral blood biomarkers (TNF-α, ROS and ICAM) and neurocognitive performance (IQ and GCS) were found to be key factors for predicting LTL across T2DM and severe mental disorders, regardless of GMS. Specifically, the proinflammatory state, adhesion molecule activity and IQ were positively associated with LTL, whereas oxidative stress and GCS were negatively associated with LTL.

A growing body of evidence supports the idea that epigenetic dysfunction is a critical component of brain development and disease pathogenesis^50^. Our results suggest interplay between epigenetics, molecular processes and neurocognitive performance across individuals with T2DM and severe mental disorders. These findings build upon emerging evidence suggesting that epigenetic mechanisms are involved in the expression of clinical phenotypes and play an important role in the development of neurocognitive impairment related to mental and neurological disorders^51^. A recent review has also shown that epigenetic modifications are implicated in vascular and metabolic damage in diabetes, which suggests that several molecular pathways that contribute to disease progression are affected^52^. Interestingly, altered DNA methylation profiles related to insulin production, β cell secretion and insulin resistance have been suggested as one of the main factors that contribute to the pathogenesis of T2DM^53^. Emerging translational evidence suggests that epigenetic mechanisms contribute to exacerbating the pathological processes that involve inflammatory responses, and consequently, they play a critical role in preserving neurocognitive and functional performance^54^. Moreover, data reflecting epigenetic mechanisms can be useful in predicting increased risk of several diseases^55^.

Overall, telomere dynamics have a strong impact on DNA methylation since they contribute to regulating cellular senescence, promoting chromosomal stability, and preventing disorder vulnerability^56^. Understanding the significance of the molecular and neurocognitive processes that lead to LTL can elucidate epigenetic mechanisms underlying T2DM and psychiatric disorders. Chronic inflammation and oxidative stress accumulation modulate LTL homeostasis and integrity, affecting multiple pathways of DNA methylation in these disorders^57^. In this regard, a healthy lifestyle is considered a potential epigenetic modulator with anti-inflammatory effects^58^. Specifically, regular physical exercise has an important role in LTL maintenance and DNA methylation due to its ability to lower oxidative damage and inflammation. Furthermore, our findings add to the accumulated evidence suggesting that a shortened LTL is associated with a counterintuitive increase in overall neurocognitive performance^59^. Telomerase is responsible for LTL and is related to neuroprotective effects against neurocognitive impairment through the promotion and proliferation of neuronal survival. Therefore, growing evidence indicates that the activation of telomerase, which promotes certain molecular mechanisms, represents a novel interventional therapeutic approach to preserve neurocognitive performance over time. Interestingly, our results are also consistent with the transdiagnostic perspective of DNA methylation, suggesting that different levels of DNA methylation could help modulate telomere dynamics or signal the risk of developing comorbidities across disorders that share an underlying dysfunction of immune-inflammatory activity, oxidative damage, and neurocognitive performance. This supports the hypothesis that different levels of DNA methylations capture different biological mechanisms underlying telomere dynamics and diseases related to telomere dysfunction (comorbidity). Therefore, these estimators might be useful biomarkers for a specific disease phenotype. For example, neurocognitive performance possibly better captures biological processes associated with telomere dynamics than others. Associations also might be specific to a psychopathological cluster or a specific type of neurocognitive impairment^51,60^.

There were several limitations of our study. The main limitation was the method of DNA methylation measurement, which used peripheral blood cells. This data should be considered with caution since no procedure was used to correct for potential cell-type bias inherent to peripheral blood. Moreover, after a year of follow-up, moderate sample attrition was observed. This may have led to a potential bias in the retention of individuals who completed the assessments and were presumably in a better clinical condition. Notable strengths of our study should also be recognized. First, we recruited a difficult-to-reach sample of patients with T2DM and severe mental disorders; moreover, the sample was representative of the population, with equal distributions of sociodemographic and clinical characteristics across participant groups. Therefore, the confounding effects of disease chronicity, comorbidity, psychiatric symptoms, tobacco consumption and BMI on DNA methylation, peripheral blood biomarkers and neurocognitive performance were minimized. Second, this study includes a novel transdiagnostic approach and the comprehensive assessment of cognitive and functional outcomes in individuals stratified by global DNA methylation. Moreover, the longitudinal design of the study allows the assessment of a potential causal relationship between LTL, inflammation, oxidative stress and neurocognitive outcomes. Finally, the multicenter nature of the study increases the external validity of the results.

In conclusion, the observed heterogeneity of patterns of DNA methylation in individuals with T2DM and severe mental disorders seems to support the hypothesis that epigenetic dysregulation occurs in a transdiagnostic manner. Likewise, while the underlying mechanisms linking the pathophysiology of these disorders to the epigenetic response are still unknown, it is well known that chronic inflammation and oxidative damage induce epigenetic changes that alter neurocognitive and functional performance. Our results may help to elucidate our understanding of the interplay between epigenetics, biological processes, and neurocognitive performance. Future studies should focus on the specific epigenetic modifications and their relation to molecular and neurocognitive pathways with the aim of implementing and developing efficient and personalized therapeutic strategies for T2DM and severe mental disorders.

## Data Availability

The data that support the findings of this study are available on request from the corresponding author, RT-S.
